# Congenital Absence of the Navicular Bone: A Rare Cause of Adult-Acquired Flatfoot and Posterior Tibial Tendon Dysfunction

**DOI:** 10.7759/cureus.85151

**Published:** 2025-05-31

**Authors:** Israa Alhashimi, Alaa Al-Taie, Syed Alam, Renan Ibrahem Adam

**Affiliations:** 1 Radiology, Hamad Medical Corporation, Doha, QAT; 2 Radiology, Hamad General Hospital, Doha, QAT; 3 College of Medicine (CMED), Qatar University, Doha, QAT; 4 Musculoskeletal Radiology, Hamad Medical Corporation, Doha, QAT

**Keywords:** adult-acquired flatfoot, congenital absence, imaging, navicular bone, posterior tibial tendon dysfunction

## Abstract

The navicular bone is critical for foot biomechanics, stabilizing the midfoot through its articulation with the talus and cuneiform bones. Variations, including accessory ossicles, hypoplasia, and complete absence, can lead to altered foot function. We present the case of a 58-year-old female patient who experienced persistent left foot pain. Imaging revealed the congenital absence of the navicular bone, resulting in altered biomechanics, midfoot instability, flatfoot deformity, and secondary degenerative changes. Posterior tibial tendon dysfunction (PTTD), a leading cause of adult-acquired flatfoot, is exacerbated by navicular anomalies. Advanced imaging plays a critical role in diagnosis, with treatment ranging from conservative management to surgery, depending on severity.

## Introduction

The navicular bone is a crucial component of the medial longitudinal arch, serving as a keystone in foot biomechanics and stabilizing the midfoot through its articulation with the talus and cuneiform bones. Anatomical variations of the navicular bone, including accessory ossicles, morphological differences, hypoplasia, and complete absence, can significantly impact foot function and predispose individuals to various pathologies [1]. Among these variations, the complete absence of the navicular bone is an exceptionally rare entity, with only a few cases reported in the literature [2].

Accessory navicular bones, also known as os tibiale externum, are the most common variant, with a prevalence ranging from 2% to 21% in the general population [1]. While often asymptomatic, accessory navicular bones can lead to chronic pain, posterior tibial tendon dysfunction (PTTD), and secondary flatfoot deformity when symptomatic [3].

Navicular hypoplasia and aplasia are extremely rare congenital anomalies [1,2]. The complete absence of the navicular bone disrupts the normal talonavicular articulation, forcing direct contact between the talus and the cuneiforms. This anatomical abnormality alters foot biomechanics, leading to midfoot instability, progressive flatfoot deformity, and secondary degenerative changes [4]. The rarity of this condition makes it a challenging diagnosis, often requiring advanced imaging for confirmation.

PTTD is the leading cause of adult-acquired flatfoot, commonly affecting middle-aged women and individuals with obesity, hypertension, or diabetes. The condition is characterized by progressive attenuation, degeneration, and tenosynovitis of the posterior tibial tendon, leading to medial arch collapse, hindfoot valgus, and forefoot abduction [5]. Studies suggest that navicular anomalies, including absence, may contribute to PTTD by altering the mechanical efficiency of the tendon, further exacerbating dysfunction and deformity [1].

Radiological assessment is essential in diagnosing navicular bone anomalies and their complications. Plain radiographs can reveal structural variations, but magnetic resonance imaging (MRI) is superior in assessing associated soft tissue pathology, including posterior tibial tendon degeneration, tenosynovitis, and ligamentous involvement [2]. The complete absence of the navicular bone can be confirmed by MRI or computed tomography (CT) scans, which demonstrate direct talo-cuneiform articulation without any evidence of the navicular bone [3].

The prognosis of navicular bone anomalies varies depending on symptom severity and the presence of secondary complications. Conservative management, including orthotics, physical therapy, and anti-inflammatory medications, is effective in early-stage PTTD. However, in cases of severe flatfoot deformity, persistent pain, or advanced tendon dysfunction, surgical interventions such as tendon reconstruction, osteotomies, or fusion procedures may be required [5]. Understanding navicular bone variations and their clinical significance is crucial for the accurate diagnosis and appropriate management of related foot disorders. The absence of the navicular bone remains an exceedingly rare condition, and its recognition is important to prevent misdiagnosis and ensure optimal patient outcomes.

## Case presentation

A 58-year-old female patient with a history of diabetes and hypertension presented with persistent left foot pain that had been ongoing for approximately six months. The pain was localized to the midfoot and ankle region and worsened with ambulation, interfering with daily activities and mobility.

Prior to presentation, she underwent multiple conservative management strategies including rest, physiotherapy, activity modification, and the use of custom-made orthopedic insoles. She was also treated with oral analgesics, initially acetaminophen and later nonsteroidal anti-inflammatory drugs (NSAIDs), which provided only partial and temporary relief.

On clinical examination, there were tenderness over the midfoot joints, loss of the medial longitudinal arch, and pain with supination and pronation of the left foot. The right foot was clinically normal, and no deformity or tenderness was noted, confirming the unilateral nature of the symptoms and structural changes.

**Figure 1:**
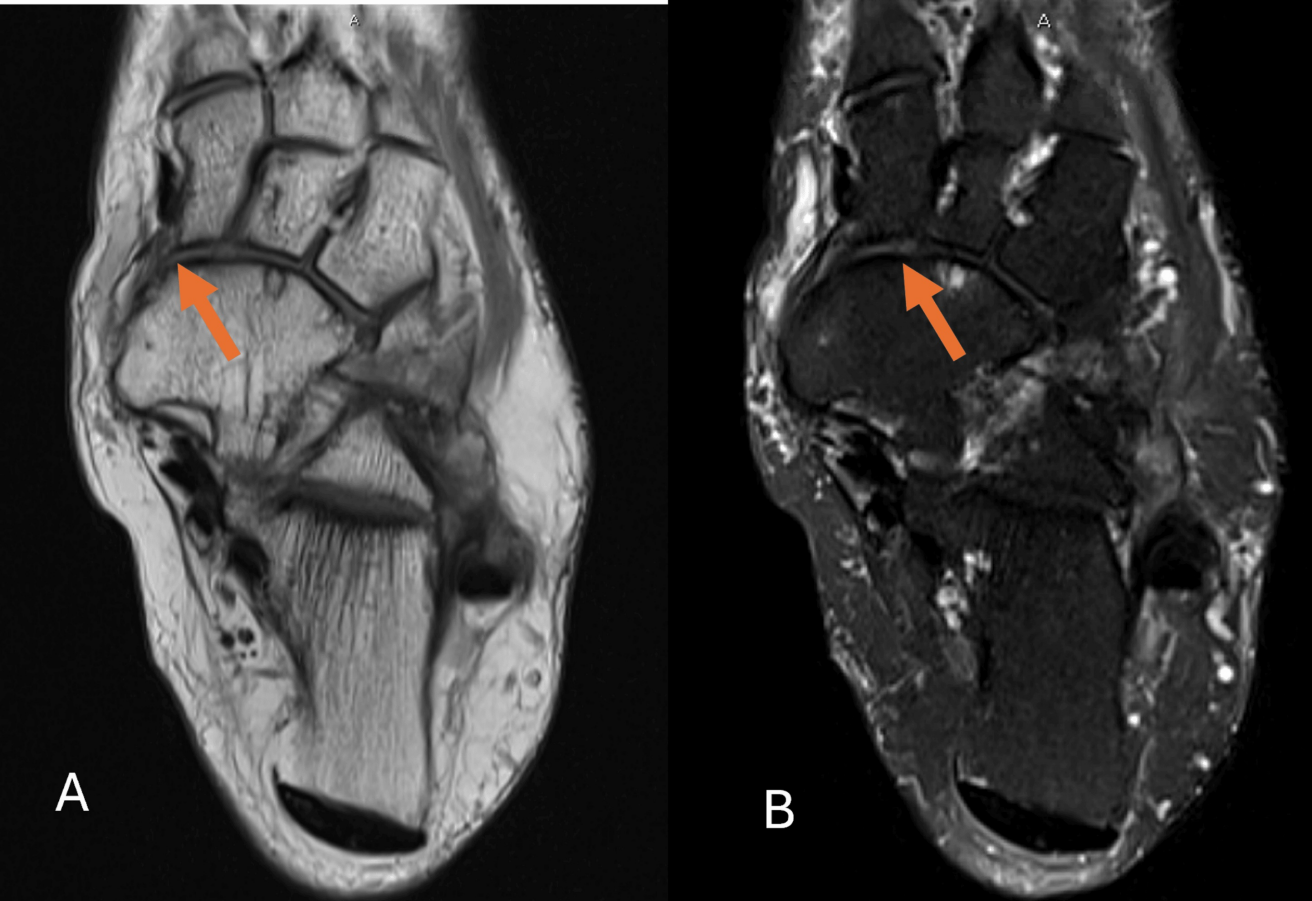
(A) Proton density axial-weighted MRI and (B) T2 fat-saturated axial-weighted MRI showing the congenital absence of the navicular bone with talo-cuneiform articulation and osteoarthritic changes (arrows) MRI: magnetic resonance imaging

**Figure 2:**
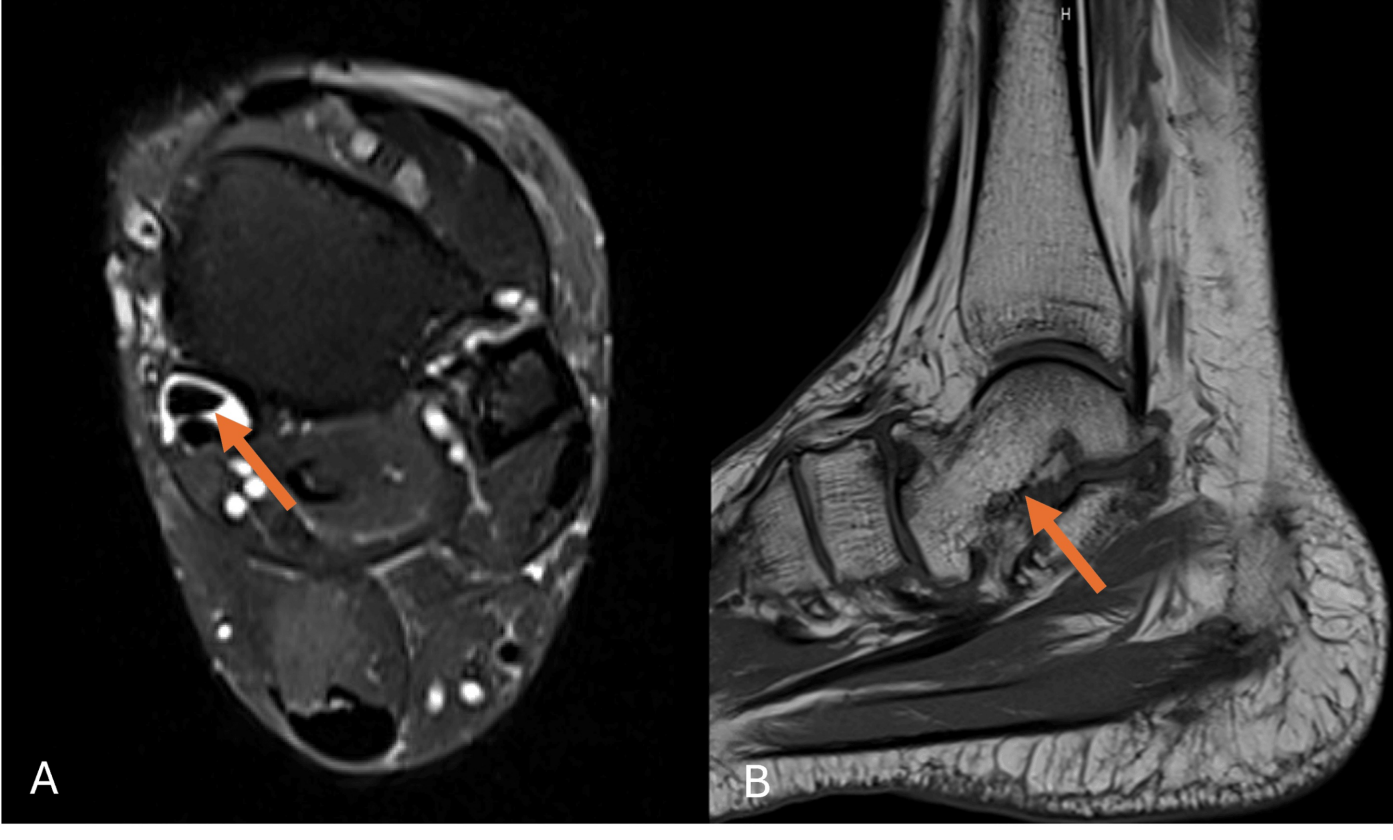
(A) T2 fat-saturated axial-weighted MRI and (B) T1 sagittal-weighted MRI (A) demonstrating posterior tibial tenosynovitis and (B) showing talar deformity and subtalar degeneration (arrows) MRI: magnetic resonance imaging

**Figure 3:**
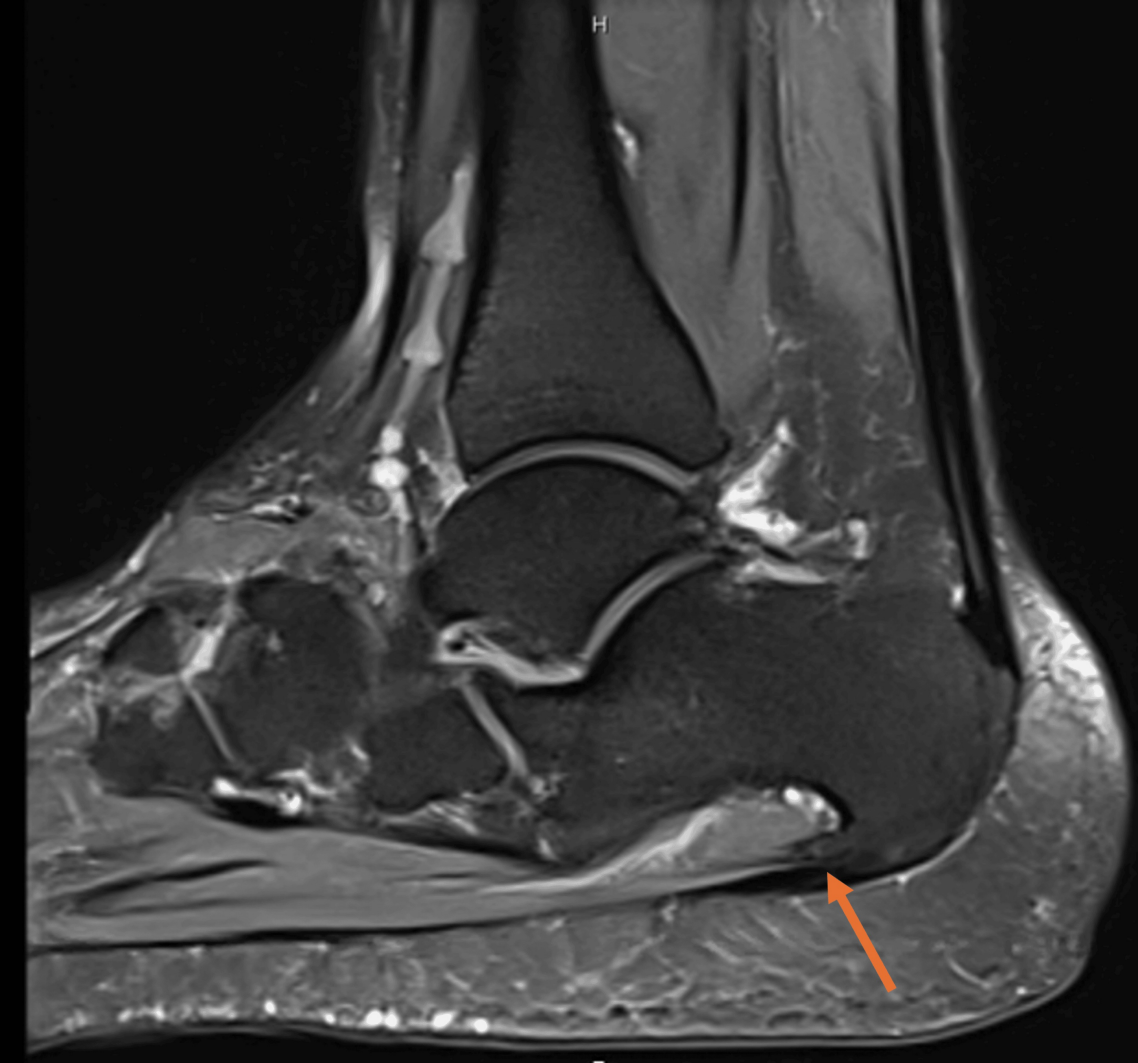
Proton density sagittal-weighted MRI revealing calcaneal spur, plantar fasciitis, and Achilles tendinopathy (arrows) MRI: magnetic resonance imaging

MRI of the left ankle and foot was performed as the initial imaging modality. It revealed the congenital absence of the navicular bone, with the talus articulating directly with the cuneiform bones, associated with osteoarthritic changes at the midfoot joints (Figure 1). Additional findings included a mild to moderate talar deformity, mild to moderate degenerative changes in the subtalar joint, and mild to moderate fluid surrounding the tibialis posterior tendon at the level of the medial malleolus, suggestive of tenosynovitis (Figure 2). There was also mild to moderate insertional tendinopathy of the distal Achilles tendon, with calcaneal insertion involvement, a large calcaneal spur, and signs of plantar fasciitis (Figure 3). The extensor, flexor, and peroneal tendons were intact. Mild to moderate subcutaneous edema was noted along the medial and lateral aspects of the ankle.

The patient was continued on conservative treatment with NSAIDs, medial arch support insoles, and activity modification. On follow-up, she reported marked symptomatic improvement and remains stable without the need for surgical intervention.

## Discussion

The presented case of a 58-year-old female patient with persistent left foot pain, characterized by the congenital absence of the navicular bone and associated osteoarthritic changes, provides a unique perspective on the interplay between navicular anomalies, PTTD, and flatfoot deformity. The congenital absence of the navicular bone is an extremely rare anomaly, with few documented cases in the literature. For instance, a case reported by Radiopaedia.org described the bilateral absence of the tarsal navicular bone, leading to secondary degenerative changes in the midtarsal joints [6]. In the current case, MRI findings demonstrate a direct articulation between the talus and the cuneiforms, leading to altered midfoot biomechanics, collapse of the medial longitudinal arch, and subsequent flatfoot deformity. This differs from more common presentations of flatfoot, such as those related to PTTD, where the medial longitudinal arch collapses due to the progressive degeneration or rupture of the posterior tibial tendon, rather than congenital structural anomalies.

PTTD is a well-recognized cause of adult-acquired flatfoot, characterized by progressive insufficiency of the tibialis posterior tendon, often leading to medial arch collapse and midfoot arthritis [7]. In the current case, MRI showed mild to moderate tenosynovitis of the tibialis posterior tendon at the malleolus, a finding consistent with early-stage PTTD. This contrasts with other reported cases of advanced PTTD, where complete tendon rupture and significant hindfoot valgus deformity are often seen [8]. Additionally, the presence of tenosynovitis in this case suggests chronic mechanical stress due to the altered biomechanics resulting from the absent navicular bone, which differs from PTTD cases caused primarily by degenerative overuse. The association between navicular anomalies and PTTD has been explored in the literature, particularly in cases of accessory navicular syndrome, where chronic stress on the tibialis posterior tendon can lead to increased pain, tenosynovitis, and secondary tendon rupture [9]. In contrast, the congenital absence of the navicular bone, as seen in this case, presents different biomechanical challenges, leading to altered load distribution across the midfoot and secondary degenerative changes without direct tendon entrapment or compression.

Other navicular anomalies, such as hypoplasia, have been reported in association with foot deformities. In a documented case of unilateral navicular hypoplasia, the condition was identified in the context of congenital talipes equinovarus, resulting in a different foot morphology compared to the current case [10]. In contrast, accessory navicular syndrome is often symptomatic due to the direct irritation of the tibialis posterior tendon at its insertion, whereas navicular absence leads to broader joint instability and degenerative progression [11]. Flatfoot resulting from trauma, degenerative changes, or inflammatory conditions may present with similar findings, but these cases often exhibit distinct underlying pathophysiology, such as joint destruction in rheumatoid arthritis or ligamentous damage in post-traumatic cases [12]. Inflammatory arthritis-related flatfoot typically presents with periarticular erosions and synovitis on MRI, which are absent in the current case [13]. Similarly, post-traumatic flatfoot often involves fracture-related joint malalignment, a key differentiating factor from congenital navicular absence.

Radiological findings in the current case show a congenital structural abnormality, secondary degenerative joint changes, and mild tenosynovitis of the tibialis posterior tendon. This is distinct from advanced PTTD cases, where marked tendon atrophy, complete rupture, and severe hindfoot valgus are common [8]. While radiological imaging provides critical insights into the structural abnormalities associated with flatfoot, orthopedic management emphasizes clinical correlation with symptoms and functional impairment to guide treatment strategies [14]. Conservative management, including orthotics, physical therapy, and NSAIDs, remains the first-line treatment for flatfoot and mild PTTD, while surgical options are reserved for cases with progressive deformity or tendon rupture. In the current case, treatment may involve supportive bracing and pain management due to the chronic nature of the structural anomaly and associated degenerative changes. This is different from cases where surgical intervention, such as tendon transfer or medial column stabilization, may be necessary for progressive PTTD or severe deformity.

## Conclusions

The congenital absence of the navicular bone is an exceedingly rare anomaly that poses significant challenges in diagnosis and management. This case highlights the impact of this condition on foot biomechanics, leading to altered load distribution, midfoot instability, and progressive flatfoot deformity. The role of advanced imaging, particularly MRI and CT, is crucial in confirming the diagnosis and identifying associated complications such as PTTD. While conservative management is effective in early-stage cases, surgical intervention may be required for severe deformities or tendon dysfunction. Understanding the pathophysiology of navicular anomalies is essential for providing optimal care and preventing misdiagnosis in patients with foot disorders.
